# Smart Governance: A Foundation for Good Governance for Health

**DOI:** 10.34172/ijhpm.8924

**Published:** 2025-04-21

**Authors:** Omid Parvizi, Azam Raoofi, Amirhossein Takian

**Affiliations:** ^1^Center of Excellence for Global Health (CEGH), Department of Global Health and Public Policy, School of Public Health, Tehran University of Medical Sciences (TUMS), Tehran, Iran.; ^2^Department of Health Management, Policy & Economics, School of Public Health, Tehran University of Medical Sciences (TUMS), Tehran, Iran.; ^3^Health Equity Research Centre (HERC), Tehran University of Medical Sciences (TUMS), Tehran, Iran.

## Dear Editor,

 The digitization of government services and the growing complexity of policy challenges^1^ necessitate innovative digital solutions to enhance governance, improve health quality, achieve universal health coverage, reduce inequalities, and increase accountability.^2^ An integrated approach in modern health information systems and medical informatics is essential, with smart governance playing a crucial role in effectively incorporating artificial intelligence (AI) innovations. This approach takes into account various social, economic, political, and cultural factors. However, challenges arising from the adoption and use of complex information and communication technologies^3^ can slow down the process.

**Figure 1:**
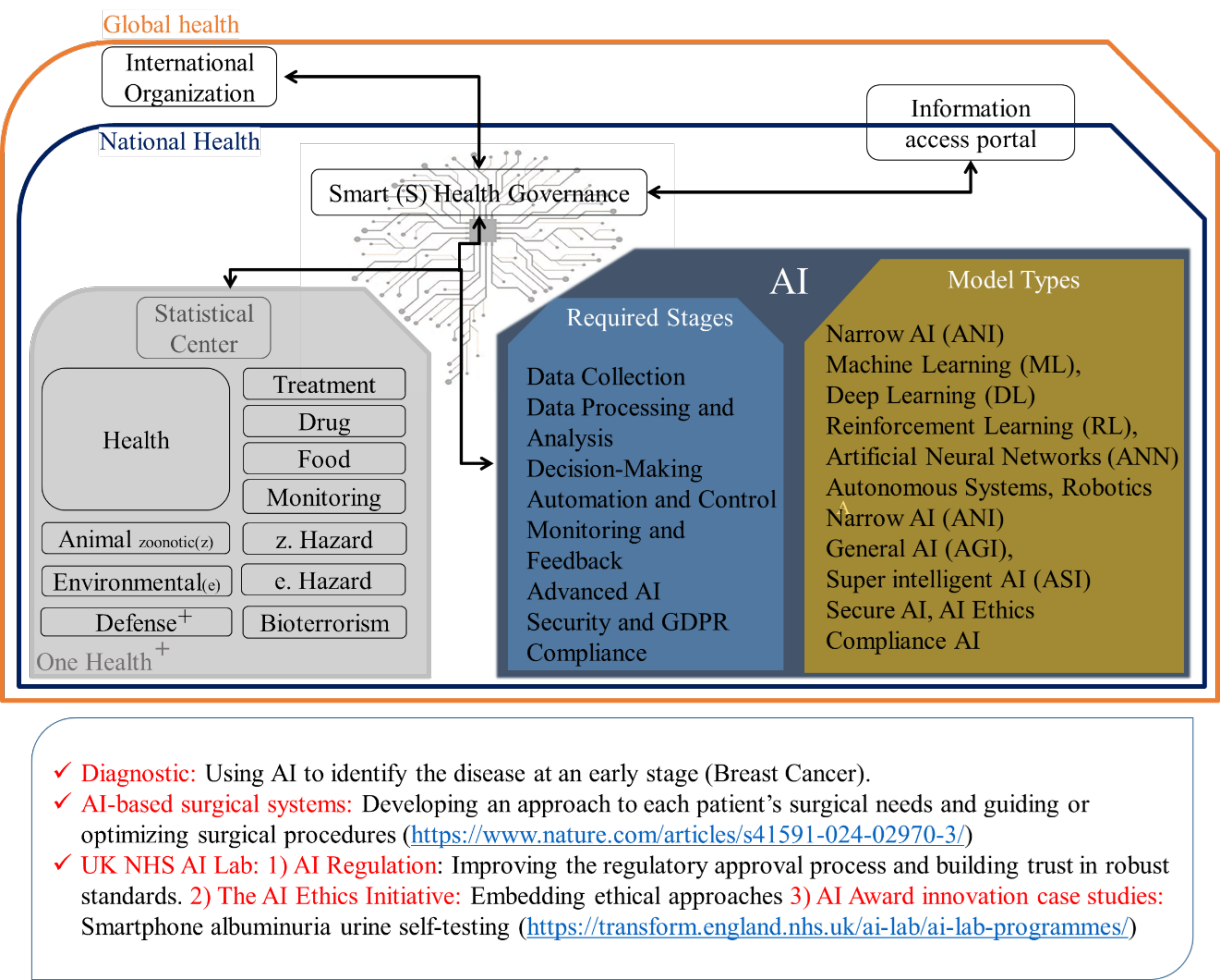
The Basic Components of Healthcare Governance and the Complexity of the Relationships Between Them. Below the figure are some real-world examples of how AI is being used to decode this idea (Source: Authors). Abbreviations: GDPR, General Data Protection Regulation; AI, artificial intelligence.

 Over the past two decades, the advancement of remote data transmission and AI have set the stage for a reevaluation of governance, introducing the concept of smart/intelligent governance.^4,5^ The current global landscape reveals the need for successful digital health initiatives to be driven by strong governance structures, which is a necessary step when deploying AI machines. The rapid development and use of AI brings significant regulatory benefits to society, bolstering the capabilities and security of governments through e-government.^6^ A hybrid relationship between AI, e-governance and data security leads to more efficiency in health governance (Figure 1). This aligns with the World Health Organization (WHO) statement on governance for health that describes: “*the efforts of governments and other actors to steer societies, entire countries or even groups of countries towards health as an integral part of well-being*”^7^ or “*AI is already playing a role in diagnosis and clinical care, drug development, disease surveillance, outbreak response, and health systems management … The future of healthcare is digital, and we must do what we can to promote universal access to these innovations and prevent them from becoming another driver for inequity*” (Tedros Adhanom Ghebreyesus, WHO Director-General).

 Strategies working across multiple health priorities should be underpinned by standards and an architecture that enables such integration. The integration of innovation within healthcare is critical for the next generation medical systems.^3^ AI-based automation, combined with smart data and cognitive computing, brings significant value to healthcare. This happens through enhancing data-driven decision-making, improving compliance, security, resource allocation, and operational efficiency, by automating administrative tasks and streamlining processes. According to the law of requisite diversity, effective regulation requires a system with a sufficient range of measures to handle various disturbances. To manage the complex processes of change, a reasonable match between the complexity of the governance system and the outside world is crucial.^8^ Frameworks like the European Commission’s AI Ethics Guidelines provide a structured approach to ensuring the use of AI and can help mitigate the associated risks.

**Figure 2:**
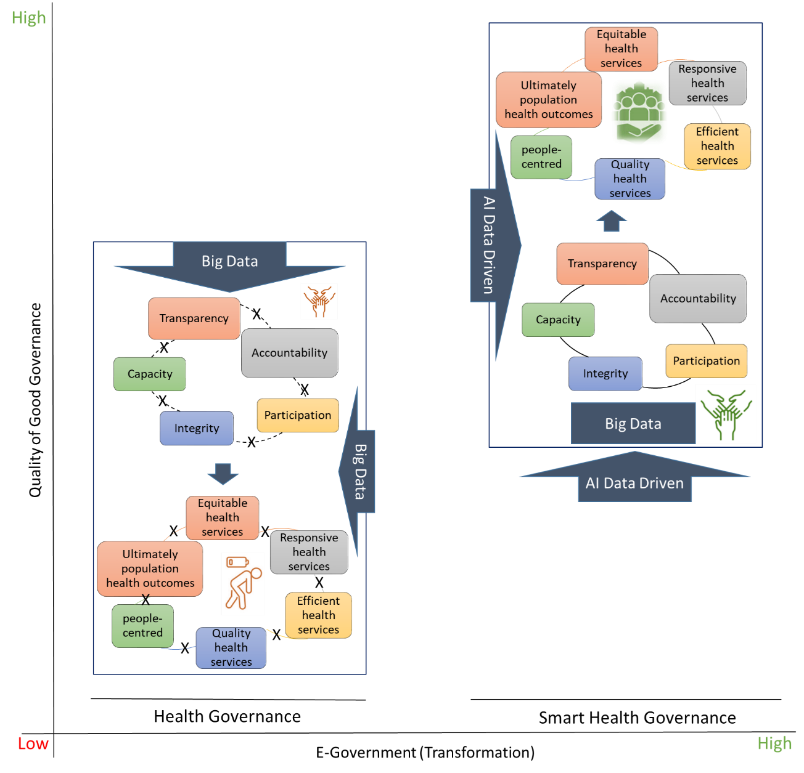
The Causal Regulatory Cycle of Good Governance and the Impact of AI Data Driven on Leveraging Big Data to Achieve Higher Quality and Create a New Concept in Governance. Source: (Authors). Abbreviation: AI, artificial intelligence.

 Smart healthcare governance will transform many aspects of the healthcare system, shaping the impact of technological developments on the future state and administration. It stabilizes the management of complexity and administrative sophistication, bringing together prominent scholars to explore novel multilevel governance challenges posed by dynamic and complex social-ecological systems (Figure 2). It also holds great promise as it enables a more refined understanding of the dynamics of rapid, interlinked and multiscale change, such as more personalized and efficient health services, predictive analytics for better resource management, and greater patient engagement through AI-driven health monitoring tools.

 AI provides tools and techniques that assist policy-makers in analyzing vast amounts of data, identifying patterns, and generating insights for evidence-based decision-making.^9^ In policy-making, AI can streamline administrative processes, improve service delivery, and optimize resource allocation. Machine learning algorithms can analyze large datasets and extract valuable information, enabling policy-makers to identify trends, anticipate future scenarios, and develop proactive strategies. Natural language processing algorithms can automate the analysis of public opinion, social media data, and public sentiment, providing policy-makers with real-time feedback and a deeper understanding of citizens’ needs and preferences.^10^

 Digital coordination and transaction platforms are increasingly replacing traditional public intermediaries as trusted third parties, thereby reshaping the organizational dynamics of collective action. Research into human decision-making behavior and the availability of real-time information promote the definition of public programs based on actual rather than the assumed behavior of social actors. AI systems increasingly support professional and unbiased management of discretionary issues by public decision-makers, enabling value-added potential, such as supplementing staffing levels, increasing productivity, allocating resources more efficiently, and promoting innovation^11^ to achieve social equity.

 The implementation of AI-based governance is a unique proposition for tackling the future healthcare system complexities and creating advanced governance. Therefore, a commitment is needed to seize the opportunity to modernize and innovate the governance system.^12^ By investigating the causes of complexity and adapting responses, AI can play a vital role in policy-makers’ decision-making for macro and micro policies.

## Ethical issues

 Ethical approval for this study was obtained from the TUMS research ethics committee (Approval number: IR.TUMS.SPH.REC-1403.259).

## Conflicts of interest

 Authors declare that they have no conflicts of interest.
